# Sequence-Based Antigenic Analyses of H1 Swine Influenza A Viruses from Colombia (2008–2021) Reveals Temporal and Geographical Antigenic Variations

**DOI:** 10.3390/v15102030

**Published:** 2023-09-30

**Authors:** Andres F. Ospina-Jimenez, Arlen P. Gomez, Maria A. Rincon-Monroy, Lucia Ortiz, Daniel R. Perez, Mario Peña, Gloria Ramirez-Nieto

**Affiliations:** 1 Grupo de Investigación en Microbiología y Epidemiología, Facultad de Medicina Veterinaria y de Zootecnia, Universidad Nacional de Colombia, Bogotá 111321, Colombia; anfospinaji@unal.edu.co (A.F.O.-J.); apgomezr@unal.edu.co (A.P.G.); marinconm@unal.edu.co (M.A.R.-M.); 2 National Veterinary Diagnostics Laboratory, Colombian Agricultural Institute (ICA), Bogotá 110931, Colombia; 3 Poultry Diagnostic and Research Center, Department of Population Health, College of Veterinary Medicine, University of Georgia, Athens, GA 30602, USA; lucia.ortizbatsche@uga.edu (L.O.); dperez1@uga.edu (D.R.P.); 4 Asociación Colombiana de Porcicultores Porkcolombia—FNP, Bogotá 111311, Colombia; mpena@porkcolombia.co

**Keywords:** antigenic drift, bioinformatics, zoonotic virus, Alphainfluenza virus, Colombia

## Abstract

Swine influenza is a respiratory disease that affects the pork industry and is a public health threat. It is caused by type A influenza virus (FLUAV), which continuously undergoes genetic and antigenic variations. A large amount of information regarding FLUAV in pigs is available worldwide, but it is limited in Latin America. The HA sequences of H1 subtype FLUAV-positive samples obtained from pigs in Colombia between 2008–2021 were analyzed using sequence-based antigenic cartography and N-Glycosylation analyses. Of the 12 predicted global antigenic groups, Colombia contained five: four corresponding to pandemic strains and one to the classical swine H1N1 clade. Circulation of these clusters was observed in some regions during specific years. Ca2 was the immunodominant epitope among Colombian viruses. The counts of N-Glycosylation motifs were associated with the antigenic cluster ranging from three to five. The results show for the first time the existence of antigenic diversity of FLUAV in Colombia and highlight the impact of spatial and temporal factors on this diversity. This study provides information about FLUAV variability in pigs under natural conditions in the absence of vaccination and emphasizes the need for surveillance of its phylogenetic and antigenic characteristics.

## 1. Introduction

Swine influenza is a contagious respiratory disease of pigs that affects the pork industry globally and poses a continuous threat to public health. It is caused by the Alphainfluenzavirus (FLUAV) of the *Orthomyxoviridae* family [1]. FLUAV has a genome comprising of eight negative-sense RNA segments and is further classified into subtypes based on two major surface glycoproteins: Hemagglutinin (HA) and Neuraminidase (NA). At least 18 HA (H1–H18) and 11 NA (N1–N11) subtypes are recognized, all detected in wild aquatic bird species (Anseriformes and Charadriiformes), except for subtypes H17N10 and H18N11, identified in fruit bats in Guatemala and Peru, respectively; and H9N2-like FLUAVs, identified in fruit bats in Egypt and South Africa [2,3,4,5]. 

FLUAV has established permanent lineages in pigs worldwide. Established subtypes are H1N1, H1N2, and H3N2, each of which show significant phylogenetic and antigenic diversity, reflecting their ancestral origin and subsequent independent evolution [6,7,8,9]. Three H1 and four H3 swine lineages have been recognized, namely H1 1A (classical), 1B (human-like), and 1C (Euro-Asiatic or Avian-like) and H3 1970, 1990, 2000, and 2010-like strains [8,9]. 

Pigs are susceptible to FLUAVs of human and avian origin because of characteristics of their respiratory tract. These include the expression of receptors for human and avian origin FLUAVs (α2,6Gal and 2,3Gal, respectively) and supportive proteins needed for viral replication (swANP32A and swANP32B) [10,11,12]. Pigs are considered “mixing vessels” or intermediate hosts where FLUAVs from different origins can reassort, evolve, and potentially acquire mammalian adaptations or new genomic constellations, as observed during the emergence of the H1N1 pandemic virus in 2009 (clade 1A.3.3.2) [13]. 

FLUAV in swine undergoes continuous evolutionary changes owing to immunological selection pressures, resulting in antigenic drift [14,15,16]. These changes primarily occur in the globular region of the immunodominant glycoprotein HA, among which the epitopes and antigenic sites of both H1 and H3 have already been identified [17,18,19]. Even though selection pressure acting on swine populations is lower than that in humans [20], global studies indicate that it has been enough for the emergence of relevant antigenic diversity with numerous antigenic clusters reported in Europe, North America, and Asia [7,21,22]. The emergence of these new antigenic clusters is considered a potential risk to human health [21,22,23]. In Latin America, the antigenic characteristics and description of antigenic clusters of FLUAV in swine have only been performed in Chile, where different antigenic variants and new clusters were identified [24]. 

In Colombia, characterization of FLUAV in pigs has been limited to phylogenetic descriptions and serological surveillance reports. Epidemiological studies based on serology indicated that both H1N1 and H3N2 have been circulating among pig herds for at least 50 years [25]. More recently, research studies led to the isolation, sequencing, and partial genetic characterization of H1N1 strains in the country [26,27,28,29]. Regarding the H3N2 subtype, neither molecular evidence nor sequence data have yet been obtained. Consequently, knowledge about the virus is limited to the H1 subtype, specifically the 1A lineage represented by the classical 1A.1 (α-H1) and pandemic 1A.3.3.2 clades [26,27,29]. Evidence of these two clades suggests the existence of at least two antigenic clusters. However, it is possible that the virus has been undergoing antigenic drift, potentially leading to the emergence of antigenic variants and clusters that differ from those found in other countries, suggesting its independent antigenic evolution across the territory. This could be the result of different acting selective pressures and lack of vaccination against FLUAV in Colombia [26].

Therefore, this study aimed to evaluate the antigenic characteristics of swine H1 FLUAV in Colombia, estimate its antigenic variation, and describe potential antigenic clusters between 2008 and 2021. This study presents for the first time the results of antigenic characterization of swine H1 FLUAV in the country, which contributes to the understanding of the antigenic evolution of the virus under natural selective pressure in the absence of vaccination. These results also highlight the importance of evaluating the antigenic characteristics of FLUAV in addition to phylogenetic analysis because of the underestimation of point mutations in antigenic regions.

## 2. Materials and Methods

### 2.1. Viruses

**Figure 1:**
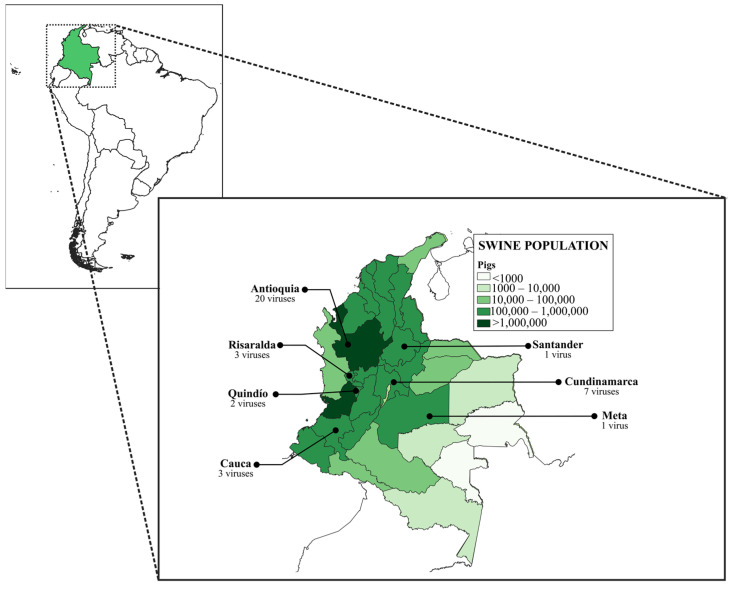
Geographic origin of influenza A virus included in this study. All viruses were from regions with high swine population density.

In this study, 37 full-length sequences of the HA gene of swine H1 FLUAVs belonging to the viral repository of the National Veterinary Diagnostic Laboratory of the Colombian Agricultural Institute (LNDV-ICA) and the Molecular and Virology Laboratory of the Universidad Nacional de Colombia (LBMV-UN) were used (Table 1). The viruses were obtained from nasal swabs and lung tissues of commercial pigs from high-density swine population regions of Colombia between 2008–2021 (Figure 1). Samples were collected during surveillance activities carried out by the LNDV-ICA and research projects conducted by the LBMV-UN. The procedures and conditions used to obtain samples from the LBMV-UN repository were approved by the Bioethics Committee of the School of Veterinary Medicine and Animal Science of the Universidad Nacional de Colombia. 

### 2.2. Phylogenetic Characterization of the HA Glycoprotein

Nucleotide sequences of the HA gene of the viruses used in this study were previously acquired by next-generation sequencing (NGS) at The University of Georgia, USA. Used sequences were deposited in the GISAID database (Table 1). For the analysis, these sequences were translated into amino acids using the SeqBuilder Pro^TM^ Software v17.0 (DNASTAR Lasergene Inc, Madison, WI, USA). The protein sequences were aligned along with 61 H1 representative strains encompassing the three swine FLUAV lineages encompassing 1930 to 2021 and six seasonal human FLUAVs. Representative sequences were collected from the protein database of the National Center for Biotechnology Information (NCBI) (https://www.ncbi.nlm.nih.gov/protein/; accessed on 13 January 2023) and are listed in Table S1. The alignment was performed with the MUSCLE-5 algorithm using the MUSCLE v5 tool [30]. The phylogenetic tree was constructed by the maximum-likelihood method using the ultrafast bootstrap approximation implemented in the IQ-TREE 1.6.12 software on a base of 1000 replicates [31,32]. The tree was edited using Interactive Tree of Life (iTOL; http://itol.embl.de; accessed on 10 February 2023) version 6.7.5. 

### 2.3. Antigenic Characterization

Antigenic characterization was performed using the sequence-based antigenic cartography method developed by Anderson et al. [33] for H1 FLUAV. This method allows for the estimation of the antigenic distances (AD) between H1 viruses based on the amino acid differences in the five antigenic epitopes of the HA. For this analysis, protein sequences were edited and adjusted for HA1 numbering [34]. Subsequently, amino acids in the five major epitopes (Sa: 124, 125, 153–157, 159–164; Sb: 184–195; Ca1: 166–170, 203, 204, 205, 235–237; Ca2: 137–142, 221, 222; Cb: 70–75) were extracted using the Extractseq tool v6.6.0.0 from the European Molecular Biology Open Software Suite (EMBOSS) (https://www.bioinformatics.nl/cgi-bin/emboss/extractseq; accessed on 5 March 2023). Based on extracted peptides, an AD matrix was constructed by calculating and averaging the five epitopic distances (ED) between each virus using the *cultevo* v1.0.2 package in the RStudio^®^ Software v4.3.1. The calculated ADs were represented in antigenic unities (AU), which are linearly correlated with the gold standard hemagglutination inhibition assay (HI) and suggest the existence of overlap recognition by antibodies at AD < 8.0 AU [33]. To infer antigenic clusters, a hierarchical clustering analysis was performed using the package *stats4* v4.3.1 in the RStudio^®^ Software. The same sequences used in the phylogenetic characterization were included in the antigenic cartography.

Antigenic maps were generated by applying a dimensional reduction to the AD matrix using the classical multi-dimensional scaling (MDS) of the *stats4* package in the RStudio^®^ Software. The optimal dimensional representation was chosen based on goodness-of-fit (GOF) calculations for dimensional spaces between 1 and 10, and the number of viruses to be represented in each map. Potential antigenic clusters were inferred based on the AD values observed between the classical and pandemic clades of the 1A lineage, and the clustering pattern observed in the hierarchical dendrograms. A K-value of 12 was selected to achieve higher discrimination resolution among both phylogenetic groups. The clusters were represented in a three-dimensional map, and the Colombian clusters in a two-dimensional map.

### 2.4. Epitope Analyses 

The impact of each epitope on the antigenic clustering pattern was evaluated using median-joining network (MJN) analysis. For this purpose, amino acid consensus of the epitopes in each antigenic cluster was implemented. Consensuses were obtained using the Cons Tool from the EMBOSS v6.6.0.0 (https://www.ebi.ac.uk/Tools/msa/emboss_cons/; accessed on 8 March 2023). Networks for the epitopes were constructed using the NETWORK v10.2.0.0 tool (https://www.fluxus-engineering.com; accessed on 14 March 2023). 

### 2.5. N-Glycosylation Analyses 

The presence of N-glycosylation motifs (NxS/T; where x is any amino acid but P) in the sequences was evaluated using the NetNGlyc-v1.0 tool from the Technical University of Denmark (DTU) (https://services.healthtech.dtu.dk/service.php?NetNGlyc-1.0; accessed on 2 April 2023). In this analysis, the entire amino acid sequence of each HA (HA1 numbering) was used. Only motifs with an N-glycosylation potential > 0.5 were considered as potentially modifiable sites.

## 3. Results

### 3.1. Colombian Swine H1 FLUAVs of the 1A.1 Clade Remain Genetically Stable, Whereas the 1A.3.3.2 Clade Shows Phylogeographic Divergence

**Figure 2:**
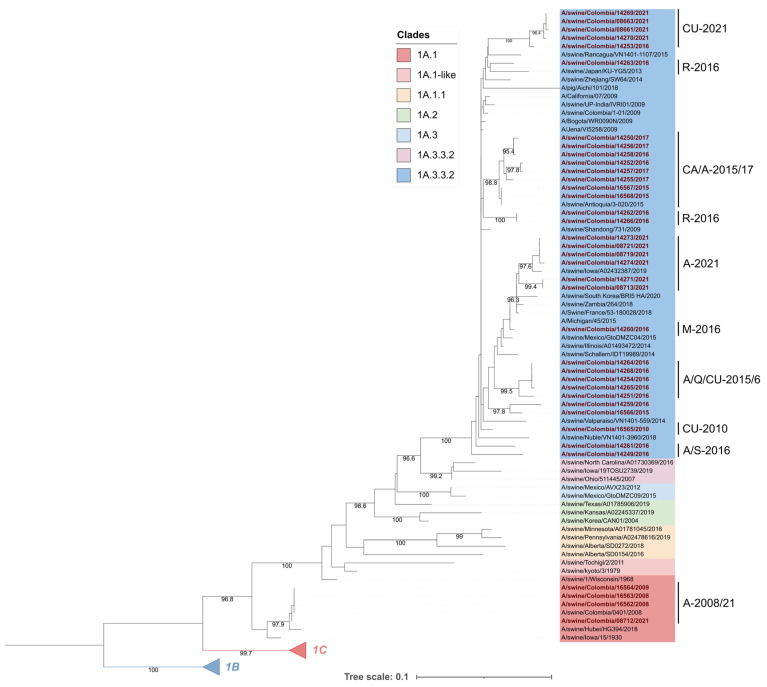
Phylogenetic tree of the HA glycoprotein of swine influenza A viruses included in this study. Colombian viruses are labeled in red and highlighted in bold. Viruses were allocated into two clades corresponding to pandemic 1A.3.3.2 and classic 1A.1. Subclades where Colombian viruses were allocated are named according to the region and years of their detection, A: Antioquia; S: Santander; CU: Cundinamarca; Q: Quindío; M: Meta; R: Risaralda; CA: Cauca.

Phylogenetic analysis showed that Colombian viruses included in the study were grouped in the 1A lineage; 4 corresponded to the 1A.1 (α-H1) clade and 33 to the 1A.3.3.2 pandemic clade. The viruses in clade 1A.1 were placed into an early divergent branch into the lineage. These viruses were closely related to each other and constituted a monophyletic group, along with one strain from Asia (A/swine/Hubei/HG394/2018) and the ancient swine FLUAV A/swine/Iowa/15/1930 from North America (Figure 2). 

Colombian viruses from clade 1A.3.3.2 had significant phylogenetic diversity. The FLUAVs from 2016 were the most diverse, as they were grouped into seven different subclades, one of which was a monophyletic group that differs from all the other viruses included in our analysis. On the other hand, viruses from 2015–2017 also displayed high phylogenetic diversity and were distributed intermixed into many subclades. 

A regional trend in FLUAVs was observed since 2015, as strains from this year showed phylogenetic divergence according to their geographical origin. This tendency was also observed in the phylogenetic grouping of viruses in 2021 (Figure 2).

### 3.2. Sequence-Based Antigenic Cartography Shows the Relatedness with Phylogeny, Geographic Origin, and Temporal Factors

**Figure 3:**
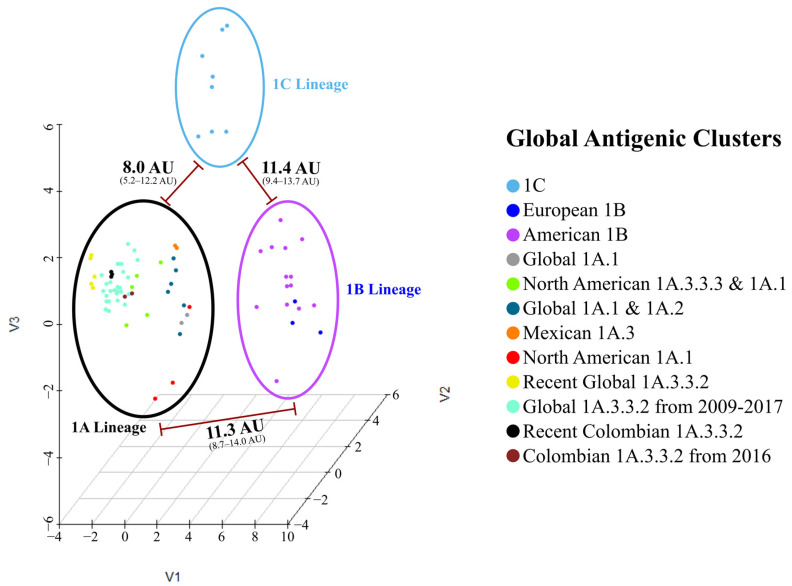
Sequence-based antigenic cartography of Colombian and global swine H1 influenza A virus. The figure shows three major antigenic groups corresponding to swine H1 lineages. The mean calculated antigenic distances between groups are shown. Viruses analyzed are represented as points colored according to the assigned antigenic group.

The sequence-based antigenic 3-D map showed three primary groups, each corresponding to one of the H1 Lineages. The 1A lineage exhibited the highest diversity comprising nine antigenic clusters, whereas lineages 1B and 1C were represented by two and one antigenic clusters, respectively. Each predicted cluster was named based on its phylogenetic, geographical, and temporal origin (Figure 3). 

**Figure 4:**
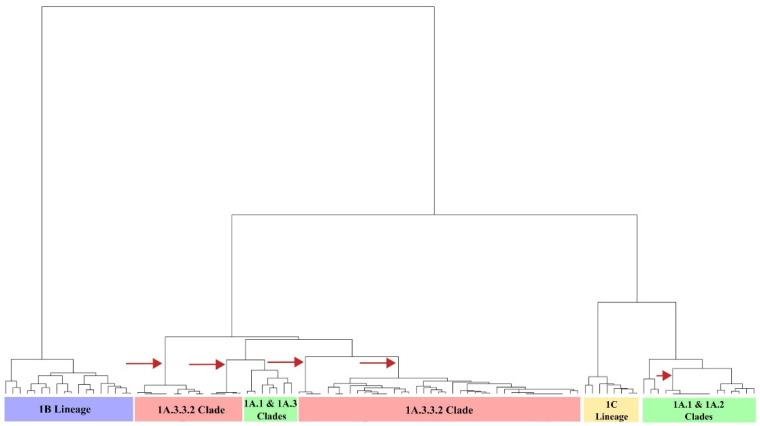
Antigenic dendrogram of the swine H1 FLUAVs analyzed. Arrows show the branches in which Colombian isolates were allocated.

Among the sequences, the mean antigenic distance was 6.8 AU. The highest (14.0 AU) was found between recent Colombian isolates (08713/21/A and 14271/21/A) and the 1B European strain A/swine/Bakum/1832/2000. The lowest was 0.0 AU and was observed between FLUAVs with a similar geographic and/or temporal origin, as can be seen in the clustering pattern and the AD matrix (Figure S1). Hierarchical analysis showed that 1B Lineage was the most antigenically divergent group among the swine H1 FLUAVs, as it was positioned in a separated branch in the dendrogram and in a distant cluster in the antigenic maps. The 1A and 1C lineages were not strongly discriminated, and both were found to be mixed in the antigenic dendrogram. Colombian swine H1 FLUAVs included in this study were distributed across five branches that corresponded to five antigenic clusters, as shown in Figure 3 and Figure 4.

### 3.3. There Were at Least Five Antigenic Clusters Distributed in Different Regions of Colombia during Specific Years

The 1A lineage contained five antigenic clusters related to classical FLUAV and four to the pandemic 1A.3.3.2 clade (Figure 4 and Figure S2). The mean AD within lineage 1A was 4.3 AU (0.0–10.0 AU).

**Figure 5:**
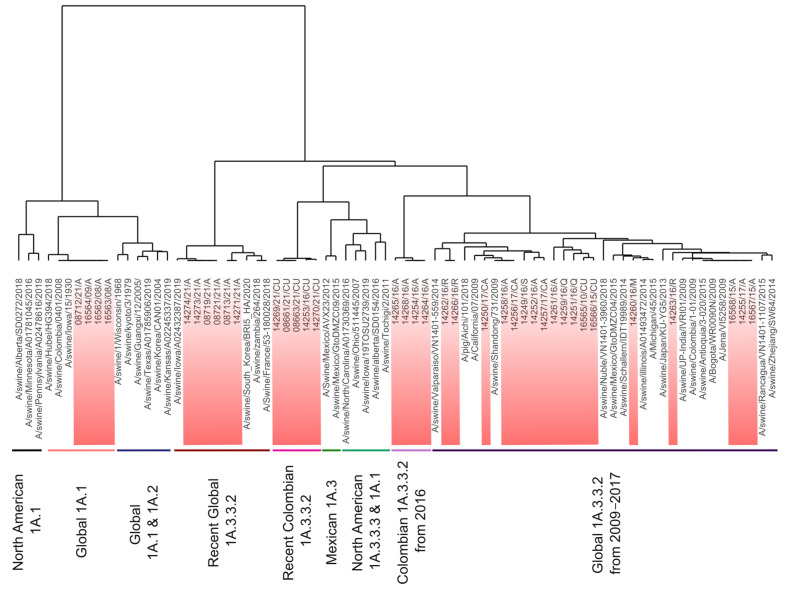
Antigenic dendrogram and clusters of the H1 1A lineage of the swine influenza A virus. Among the nine clusters, five corresponded to pandemic 1A3.3.2 and four to classic clades. Colombian viruses are highlighted in red.

The classical clusters were Global 1A.1 (G1A.1), North American 1A.3.3.3 and 1A.1 (N1A.1–3), North American 1A.1 (N1A.1), Mexican 1A.3 (M1A.3), and Global 1A.1 and 1A.2 (G1A.1–2). G1A.1 was formed by viruses from the classical clade 1A.1 detected in the Americas between 1930 and 2021. In this group, the ancient strain A/swine/Iowa/17/19130, all classical Colombian viruses, and a virus from China detected in 2018 (A/swine/Hubei/HG394/2018) were allocated showing high antigenic stability (Figure 5). The N1A.1–3 group was diverse (Table 2) and included viruses from North America of the 1A.3.3.3 (γ-H1) and 1A.1 phylogenetic clades. The N1A.1 cluster comprised strains of the 1A.1 phylogenetic clade detected in the second half of the 2010s. This was the most divergent cluster, exhibiting an AD of approximately 8.0 AU, which suggests a lack of cross-reactivity with the others in the lineage. M1A.3 included two viruses of the γ-H1 phylogenetic clade identified in 2012 and 2015 in Mexico, notably (6.7 AU) distinct from other 1A.3 strains. The G1A.1–2 cluster contained strains of the 1A.1 and 1A.2 (β-H1) phylogenetic clades. These were subclustered according to a temporal pattern, with strains from the 2000s being grouped apart from the older viruses (Figure 5).

The pandemic clusters were Global 1A.3.3.2 from 2009–2017 (PDM09-17), Colombian 1A.3.3.2 from 2016 (PDM-CO-16), Global 1A.3.3.2 (PDM-21), and Recent Colombian 1A.3.3.2 (PDM-CO-21).

The PDM09-17 cluster had a mean AD of 2.0 AU (0.0–5.6 AU). It comprised three vaccine strains: A/California/07/2009 and A/Michigan/45/2015, used in humans; and A/Jena/VI5258/2009, used in swine. The human strain A/Bogota/WR0090N/2009 reported in Colombia was also grouped here, and it was antigenically similar to Colombian swine viruses detected between 2009 and 2017. A Chilean strain (A/swine/Nuble/VN1401-3960/2018) was the most divergent, with an AD of 3.0 AU from the nearest virus. In this cluster, 18 Colombian viruses were identified. These had an AD of 2.1 AU (0.0–4.4 AU) among each other and were antigenically distant from viruses reported in Chile and Japan, but closer to viruses previously reported in Colombia, Europe, and Asia, as shown in Figure 5.

PDM-CO-16 cluster contained only four viruses from the Antioquia region identified in 2016. This group had a high antigenic relatedness. In the antigenic dendrograms and maps, this cluster was plotted near to the PDM09-17 (Figure 4, Figure 5 and Figure S2). However, based on the AD calculated between these two groups (3.7 AU), both were considered antigenically distinguishable. Nevertheless, a virus (14251/16/Q) from the PDM09-17 showed similarity to the PDM-CO-16 cluster by 1.7 AU. 

The PDM-21 group comprised FLUAVs from North America, Asia, and Africa detected between 2018 and 2020, as well as six Colombian viruses identified in the Antioquia region in 2021. The cluster exhibited a high antigenic relatedness with a mean AD of only 0.9 AU (Table 2). Viruses detected during 2018–2019 were more antigenically related to each other than to those detected later during 2020–2021. Colombian viruses within the cluster were antigenically related to each other having an AD only of 0.8 AU (0.0–1.5 AU). In these viruses, the existence of a North American-like antigenicity was noticed in 14274/21/A, 14273/21/A, 08719/21/A, and 08721/21/A strains being only 0.3 AU apart from A/swine/Iowa/A02432387/2019. Conversely, a Eurasian-like antigenicity was observed in 08713/21/A and 14271/21/A, which were 0.8 AU apart from A/swine/Zambia/264/2018 and A/Swine/France/53-180028/2018.

In the PDM-CO-21 cluster, only five viruses were grouped. These were identified in the Cundinamarca region, four in 2021 and one in 2016. These FLUAVs displayed considerable antigenic similarity, with only an AD of 0.2 AU (0.0–0.3 AU).

**Figure 6:**
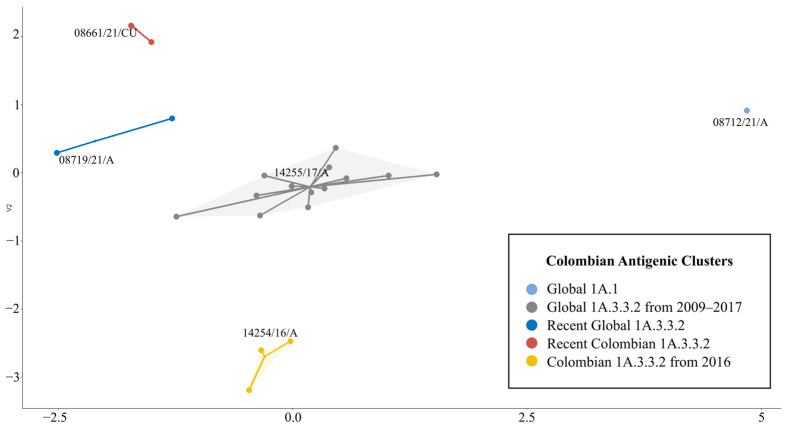
Antigenic map of the Colombian H1 swine influenza A viruses. Strains are represented as points colored interconnected according to the assigned antigenic cluster. In the groups, the names of representative strains are shown.

Due to the low resolution of the AD between PDM09-17 and PDM-CO-16 clusters in the global three-dimensional antigenic map, a two-dimensional map containing only the Colombian isolates was constructed. In this cartography, the five antigenic clusters and their AD were clearly visualized, confirming that PDM-CO-16 is a divergent group located apart from other pandemic clusters. The 2-D map showed the PDM09-17 cluster as the central group, being located near all other clusters. In addition, it showed that recent PDM-21 and PDM-CO-21 clusters, despite being antigenically different from each other, have a similar tendency. The classical G1A.1 cluster was represented as a single point in a marginal position, proving their antigenic stability and divergence from the pandemic clusters (Figure 6).

### 3.4. Antigenic Characteristics of 1B and 1C Lineages Were Partially Influenced by Phylogeny and Geographic Factors

Antigenic clusters of the 1B lineage were American 1B (1B-AM) and European 1B (1B-EU). The mean AD between both groups was 7.0 AU. 

1B-AM had an AD of 4.1 AU (0.0–7.0 AU) and included viruses from Chile, Mexico, and the USA, as well as one strain from Vietnam and two human viruses (A/Medellin/WRAIR1297P/2008 and the vaccine strain A/Brisbane/59/2007). Viruses in this cluster tended to cluster according to regional and phylogenetic patterns (Figure S3). However, a high AD was observed for some phylogeographic-related viruses. This was the case of the Chilean viruses of the 1B.2-other clade and North American strains of 1B.2.2 (Table 3). In 1B-EU, the AD was 3.1 AU (0.0–5.6 AU) and the grouping pattern appeared to be related to the year of detection (Figure S3).

In the 1C lineage, the single antigenic cluster had a mean AD of 3.0 AU (0.3–6.3 AU). In this group, antigenic divergence was also affected by the geographic origin of the viruses, with larger distances between strains from different countries. This was found in all French viruses, where an AD > 4.0 AU from viruses from other countries was consistently calculated, except for A/swine/France/Cotes_dArmor-0388/2009. A three-dimensional map of the antigenic cartography of 1B and 1C viruses is shown in Figure S4.

### 3.5. Predicted Antigenic Clusters in Colombia Carried Point Mutations whitin the Epitopes, with Ca2 Demonstrating Immunodominance

**Figure 7:**
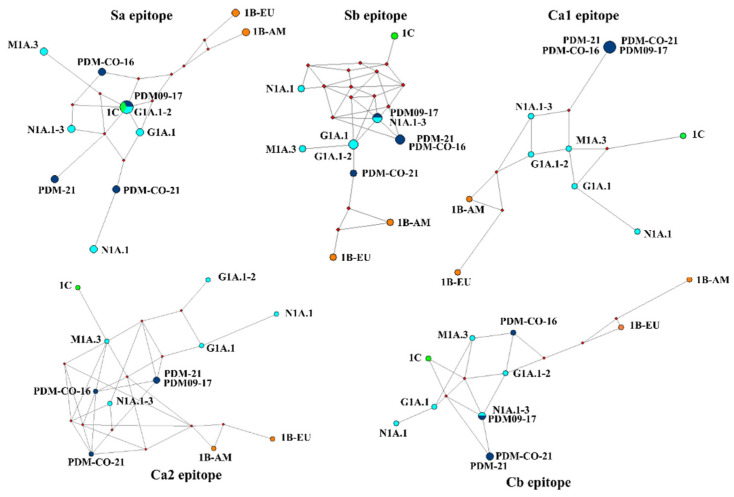
Median-joining networks of the five epitopes of swine H1 influenza A virus. Node size and color are related to the clusters and their phylogeny. The phylogeny is represented with different colors: light blue for classical clades of the 1A lineage, dark blue for the pandemic clade of the 1A lineage, orange for the 1B lineage, and green for the 1C lineage.

MJN showed that the antigenic divergence among the 12 clusters was mainly determined by the average difference among the five epitopes. None of the networks displayed a single node for each antigenic cluster. Instead, networks contained between 9 and 11 nodes, indicating that some epitopes (Sa, Sb, and Cb) were similar in certain clusters, usually among viruses of the same lineage (Figure 7). Interestingly, the Sa epitope appeared to be conserved in strains of different lineages, as two clusters of the 1A lineage (PDM09-17 and G1A.1–2) were represented as a single node along with the 1C cluster. This conservation was also observed in the Ca1 epitope, in which all the 1A.3.3.2 clusters were identical. The Ca2 epitope had the highest inter-cluster resolution, representing 11 nodes, of which only 1 was shared and contained PDM-CO-21 and PDM-21 clusters. The network of this epitope also accurately reflected the phylogenetic origin of the FLUAVs (Figure 7). The MNJ of the five epitopes confirmed the antigenic divergence of the 1B lineage, with the two clusters always located apart from each other and from the 1A and 1C lineages. 

Determination of point mutations in the epitopes of Colombian FLUAVs revealed no variations in classical viruses, which displayed 100% conservation. In contrast, pandemic viruses exhibited variations, with the Ca2 epitope demonstrating immunodominance, as indicated by its low conservation percentage. Moreover, mutational tendencies related to the geographical origin and year of detection were observed (Table 4).

### 3.6. N-Glycosylation Motifs of Colombian Swine H1 FLUAVs Varied between Three and Five and Were Related to the Predicted Antigenic Cluster

**Figure 8:**
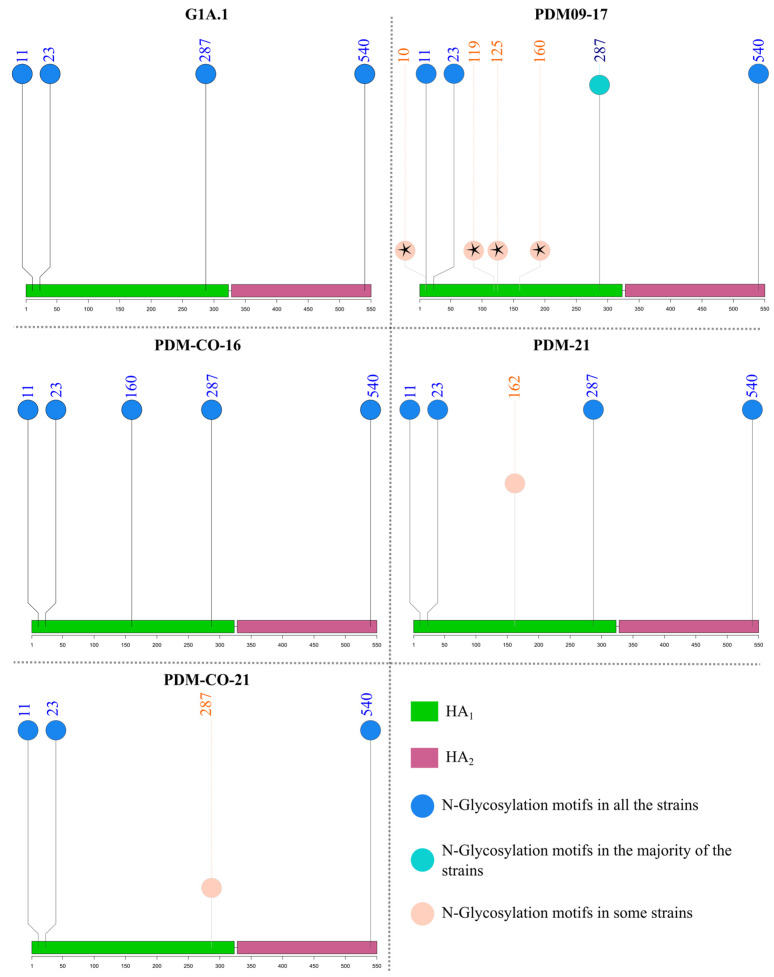
N-Glycosylation motifs in the antigenic clusters of swine H1 influenza A virus where Colombian viruses were included. The altitude of the circles represents the frequency of detection of the N-Glycosylation sites in each cluster. Motifs that were absent in Colombian viruses are marked with black stars.

Motifs predicted in Colombian sequences are summarized in Table S2. Four consistent sites of N-glycosylation motifs were found across all H1 sequences at amino acid positions 11, 23, 287, and 540, except for the 1C lineage and some 1A.3.3.2 and 1B viruses. Among classical strains, only members of the N1A.1 cluster possessed five modifiable residues (consistent sites plus 162). In the G1A.1–2 cluster, A/swine/Kansas/A02245337/2019 contains an extra site at amino acid 10, making it a unique virus with five potential N-glycosylation sites. There was no gain or loss of N-glycosylation sites in classical Colombian viruses (Figure 8).

Regarding pandemic viruses, between three and six N-Glycosylation positions were observed, with the majority possessing the four consistent sites. Strains with three and six motifs belonged to the PDM09-17 cluster. These were A/Bogota/WR0090N/2009, in which the N-Glycosylation potential at 540 was lost, and A/swine/Valparaiso/VN1401-559/2014, in which additional positions were present at amino acids 10 and 119. Viruses with five sites were members of the PDM09-17 (A/swine/Nuble/VN1401-3960/2018 and A/swine/Indiana/A02524527/2020), PDM-21 (A/Swine/France/53-180028/2018, A/swine/Zambia/264/2018, and all Colombian viruses), and PDM-CO-16 clusters. Colombian viruses from PDM-21 and PDM-CO-16 clusters had the additional motif at residues 162 and 160, respectively. Interestingly, in the PDM-CO-21 cluster, all viruses except 14253/16/CU lost one motif at position 287 (Figure 8). 

All viruses in the 1C cluster had only three motifs at positions 11, 23, and 540, except for A/swine/Finistere/2899/1982, which had four motifs, including one at position 287. 

The 1B lineage exhibited the highest motif counts, ranging from five to eight. Within 1B-EU, the four consistent sites, as well as one at 160, were displayed. The N-Glycosylation pattern of the 1B-AM cluster revealed acquisition and loss of motifs according to the phylogeny of the viruses. Five motifs were observed in most strains of the 1B.2.1 phylogenetic clade; conversely, six were frequently detected in the 1B.2.2, located at positions 11, 23, 54, 125, 160, and 540. The 1B.2-other clade had the highest N-Glycosylation motif counts, with many strains possessing up to seven sites at 11, 23, 54, 125, 160, 287, 321, and 540. All the consistent sites were present in the human viruses, which displayed additional motifs at positions 54, 125, and 160.

## 4. Discussion

In this study, we provide for the first time in silico evidence of antigenic diversity among swine H1 FLUAV in Colombia using the sequence-based antigenic cartography approach. The method allowed for the inference of 12 global antigenic clusters, 5 of which were present in Colombia, with 2 detected only in the country. These results highlight the need for permanent surveillance of the antigenic evolution of the swine FLUAV in Latin America, particularly in countries like Colombia, where the virus might evolve unnoticed as could happened with the pandemic H1N1 virus after its introduction in 2009, increasing its zoonotic and pandemic risk. 

The phylogenetic analysis of the Colombian viruses confirmed what has been previously proposed about the genetic and antigenic dominance of the pandemic clade over classical FLUAV in the country since 2008 with no indication of the introduction of new H1 lineages or phylogenetic clades [26,27,28,29]. Nevertheless, the results presented here also provide evidence of the maintenance of the classical virus in Colombian pigs until 2021. 

In this research, 4.0 AU was suggested as a potential threshold for considering two swine H1 FLUAV strains as antigenically distinguishable by the in silico method. This value is proposed based on the calculated AD between classical and pandemic viruses, supported by evidence of significant antigenic variations and slight cross-reactivity between clades [7]. Using this cut-off is supported by the findings of Anderson et al. [33] about the lack of overlapping antibodies recognition beyond an AD of 8.0 AU and the proved proportional loss of HI cross-reactivity between viruses when the highest ADs are calculated. However, it is important to note that this value must be validated through in vitro and in vivo approaches that captured the biological impact of specific mutations in the epitopes.

In Colombia, the five predicted antigenic clusters were related to the phylogeny of the viruses with one classical (G1A.1) and four pandemic (PDM09-17, PDM-CO-16, PDM-21, and PDM-CO-21) clusters. These were influenced by geographic and temporal factors. 

The first antigenic cluster detected was the classical G1A.1, which was first identified in 2008 and persisted at least until 2021. This is likely the first established cluster in Colombia, considering that early serologic evidence suggests its circulation in the Antioquia region since the 1970s [25]. The virus was probably introduced from North America during the 1900s through the movement of live animals, as occurred in Asia [6,35]. This is supported by the phylogenetic relatedness of Colombian classical viruses with the ancient swine FLUAV strain reported in North America by Shope et al. in the 1930s [36] and an Asian strain. It is remarkable that despite its circulation for over 50 years in the country, the cluster has remained antigenically intact with no observed antigenic drift or posttranslational changes. Therefore, we propose that antigenic stability is the result of several factors. First, it is plausible that the low immunological pressure in Colombian herds, due to the absence of vaccination against FLUAV in pigs, has allowed for its circulation under no antigenic selective forces. Another factor is the population dynamics in swine herds, which allows for the persistence of naïve animals where the virus can replicate without significant immune pressure [37,38,39,40]. Finally, it is possible that the classical FLUAV has been circulating in Colombia at a low level under the shadow of the immunodominant pandemic clade.

After the emergence of the pandemic 1A.3.3.2 clade, the PDM09-17 cluster was established in the country, as happened in several countries around the globe. Once in the country, it remained the immunodominant group in the evaluated regions until 2021. This cluster was first introduced into the susceptible Colombian swine population during the pandemic wave in 2009 [27], probably from human sources [41,42]. Because the introduction of the cluster occurred simultaneously in many geographic regions [42,43,44,45,46,47], the cluster contained viruses from Asia, Europe, and America. According to hierarchical analysis, some antigenic drift occurred, giving rise to two subclusters. This is consistent with previous reports on the diversification and antigenic variation of the pandemic HA during its dissemination [44,48,49,50], and accounted for the diversity observed at the intra-cluster level. Regarding the Colombian viruses within this cluster, the gain or loss of N-Glycosylation motifs were not detected. As a result, these possessed the same sites found in G1A.1, indicating relatively low immunological selection pressure among pigs in the country [51]. Concerning viruses from other countries, the gain of some N-Glycosylation motifs was noted in strains from Chile and India, probably due to their introduction into pigs after previous antigenic evolutionary steps in humans, as the gained sites have been related to the human host [51,52].

Interestingly, the pandemic PDM-CO-16 cluster cocirculated along with PDM09-17 only in the Antioquia region during 2016, and was not detected again. This group was both antigenically and phylogenetically distant from other pandemic viruses included in the study. The relationship of this cluster with 14251/16/Q within the PDM09-17 cluster suggests that PDM-CO-16 could emerge from strains of the earlier clade, and that Quindío’s strain is an intermediate antigenic variant. It is also possible that this cluster was introduced into swine populations in Antioquia from humans, as we noticed an additional N-Glycosylation mark at position 160, which has been related to human adaptation [51,52]. In addition, we found that the cluster contained variations at the amino acid level at certain positions, and the existence of the mark P137S associated with the seasonal evolutive pattern of FLUAV in humans during the end of 2015–2016 in the Northern Hemisphere [53]. However, human-to-swine spillover events in Colombia are difficult to probe due to the absence of molecular surveillance of human FLUAV in the country. 

Since 2021, two divergent pandemic clusters have appeared in the country: PDM-21 and PDM-CO-21. Both clusters were only detected in two geographically restricted swine populations in that year, with PDM-21 limited to the Antioquia and PDM-CO-21 to the Cundinamarca regions. 

Colombian viruses within PDM-21 showed antigenic profiles that were either American-like or Eurasian-like. The origin of this pattern could be related to the independent introduction of two genetically related FLUAV into pigs from different geographic sources during the international movement of animals and humans. It is probable that Colombian viruses from this cluster originated from a global human FLUAV, considering the presence of mutations K163G, E235D, and S74R, and the gain of an N-Glycosylation motif at 162 previously reported in pandemic viruses from humans [54,55,56,57]. The cluster likely reached swine populations in Eurasia and North America, from where it was then introduced to Colombia. It is possible to state this by considering the phylogenetic relatedness of Colombian strains of the cluster with FLUAVs detected in swine. Because of the observation that Eurasian-like viruses in the cluster (14271/21/A and 08713/21/A) did not have the S162N mutation that originated the additional N-Glycosylation motif, we propose that these viruses were introduced into pigs earlier than the North American-like ones.

The PDM-CO-21 cluster was entirely Colombian, and its first detection was performed in 2016. In 2021, it dominated the Cundinamarca region without evidence of antigenic drift. The apparent antigenic stability of the cluster indicates that once established in the swine population, it has been remained restricted to pigs [20]. This is supported by the high antigenic similarity between the recent isolates with the 14253/16/CU and their N-Glycosylation pattern, where recent isolates from 2021 have lost one motif at 287. This low level of posttranslational modification in HA has been associated with non-human hosts [51,58]. The origin of the cluster in the Cundinamarca region is uncertain; however, according to phylogenetic analysis, it was related to Asian, Colombian, and South American strains detected during the 2010s. This phylogenetic relatedness with FLUAVs detected in distant swine populations suggests its appearance during the frequent introduction of the pandemic clade in 2009 [41], or shortly after, during the diversification of the clade [48,49,50]. The absence of previous phylogenetic evidence of this cluster in the country could be related to low molecular surveillance, which could have allowed for its circulation to go undetected, as has been proposed in the “unsampled pig herd theory” [59].

Regarding the predicted clusters of the 1B and 1C lineages, a high mean AD (>3.0 AU) was always observed, indicating low antigenic resolution of the method implemented in those groups. We believe this could be due to two main reasons. On the one hand, it is probable that the focus of our analysis for the 1A lineage affected the separative capacity of the sequence-based antigenic cartography in the 1B and 1C lineages. On the other hand, it is also possible that the number of clusters selected to represent antigenic diversity in the 1A lineage in this study (K = 12) was insufficient for the diversity in the 1B and 1C, and a higher K-value was required. These assumptions must be evaluated and validated in future studies. 

In epitope analyses, the immunodominance among Colombian viruses was in Ca2, which is contrary to what has been reported previously in other countries where the epitopes Sa and Sb usually display major variation [60,61,62]. This could be a result of the low antigenic pressure among Colombian pigs due to the absence of vaccination that allowed for the conservation of epitopes located near the receptor-binding domain of HA. Intriguingly, antigenic conservation existed between global viruses at the Sa site of the 1A and 1C lineages. The relatedness of both groups has been previously observed using HI-based methods and is probably explained by their avian origin [7]. Considering that there is no evidence of a strong convergent evolution neither in 1A nor 1C [63], it is possible that the configuration of Sa has its origin in avian hosts, and once in swine, it has been maintained with minor changes due to a low selective pressure. However, it is necessary to consider that in the 1A lineage, the existence of an N-Glycosylation site at Sa could affect its antigenic similarity with the 1C lineage.

## 5. Conclusions

In this study we report, for the first time, evidence of antigenic drift and the existence of many potential antigenic clusters in swine H1 FLUAV in Colombia, contributing to the knowledge of antigenic evolution and diversity of the virus in Latin America and the rest of the world. The antigenic clusters found were influenced by spatial–temporal factors, as our results revealed the occurrence of independently new antigenic variants among Colombian regions. This study highlights the need for continuous surveillance and evaluation of not only the phylogenetic characteristics of swine FLUAV, but also its antigenic variations. This can be achieved by the sequence-based antigenic cartography method used here, as it represents a rapid, low-cost, low-labor, and useful tool for the study of antigenic characteristics and the selection of representative strains, which can be implemented in countries where the HI method is not an option or is not enough to provide full information about antigenic diversity. This information is essential to improve control and diagnostic strategies to minimize the health and economic impact of swine FLUAV in Colombia and the rest of the world.

## Figures and Tables

**Table 1:** Swine H1 influenza A viruses included in the study.

Virus Name	Virus ID	Year	Subtype	HA Lineage	Accession Number
A/swine/Colombia/16562/2008	16562/08/A	2008	H1N1	1A.1	EPI2719368
A/swine/Colombia/16563/2008	16563/08/A	2008	H1N1	1A.1	EPI2721678
A/swine/Colombia/16564/2009	16564/09/A	2009	H1N1	1A.1	EPI2721686
A/swine/Colombia/16567/2015	16567/15/A	2015	H1N1	1A.3.3.2	EPI2721687
A/swine/Colombia/16568/2015	16568/15/A	2015	H1N1	1A.3.3.2	EPI2721688
A/swine/Colombia/14254/2016	14254/16/A	2016	H1N1	1A.3.3.2	EPI2721689
A/swine/Colombia/14258/2016	14258/16/A	2016	H1N1	1A.3.3.2	EPI2721690
A/swine/Colombia/14264/2016	14264/16/A	2016	H1N1	1A.3.3.2	EPI2721691
A/swine/Colombia/14268/2016	14268/16/A	2016	H1N1	1A.3.3.2	EPI2721692
A/swine/Colombia/14252/2016	14252/16/A	2016	H1N1	1A.3.3.2	EPI2721693
A/swine/Colombia/14265/2016	14265/16/A	2016	H1N1	1A.3.3.2	EPI2721694
A/swine/Colombia/14261/2016	14261/16/A	2016	H1N2	1A.3.3.2	EPI2721695
A/swine/Colombia/14255/2017	14255/17/A	2017	H1N1	1A.3.3.2	EPI2721696
A/swine/Colombia/08712/2021	08712/21/A	2021	H1N1	1A.1	EPI2721697
A/swine/Colombia/08713/2021	08713/21/A	2021	H1N1	1A.3.3.2	EPI2721698
A/swine/Colombia/14271/2021	14271/21/A	2021	H1N1	1A.3.3.2	EPI2721699
A/swine/Colombia/08719/2021	08719/21/A	2021	H1N1	1A.3.3.2	EPI2721700
A/swine/Colombia/08721/2021	08721/21/A	2021	H1N1	1A.3.3.2	EPI2721701
A/swine/Colombia/14273/2021	14273/21/A	2021	H1N1	1A.3.3.2	EPI2721702
A/swine/Colombia/14274/2021	14274/21/A	2021	H1N1	1A.3.3.2	EPI2721703
A/swine/Colombia/14250/2017	14250/17/CA	2017	H1N1	1A.3.3.2	EPI2721704
A/swine/Colombia/14256/2017	14256/17/CA	2017	H1N1	1A.3.3.2	EPI2721705
A/swine/Colombia/14257/2017	14257/17/CA	2017	H1N1	1A.3.3.2	EPI2721706
A/swine/Colombia/16565/2010	16565/10/CU	2010	H1N1	1A.3.3.2	EPI2721707
A/swine/Colombia/16566/2015	16566/15/CU	2015	H1N1	1A.3.3.2	EPI2721708
A/swine/Colombia/14253/2016	14253/16/CU	2016	H1N1	1A.3.3.2	EPI2721709
A/swine/Colombia/08661/2021	08661/21/CU	2021	H1N1	1A.3.3.2	EPI2721710
A/swine/Colombia/08663/2021	08663/21/CU	2021	H1N1	1A.3.3.2	EPI2721711
A/swine/Colombia/14269/2021	14269/21/CU	2021	H1N1	1A.3.3.2	EPI2721712
A/swine/Colombia/14270/2021	14270/21/CU	2021	H1N1	1A.3.3.2	EPI2721713
A/swine/Colombia/14260/2016	14260/16/M	2016	H1N2	1A.3.3.2	EPI2721714
A/swine/Colombia/14259/2016	14259/16/Q	2016	H1N1	1A.3.3.2	EPI2721715
A/swine/Colombia/14251/2016	14251/16/Q	2016	H1N1	1A.3.3.2	EPI2721716
A/swine/Colombia/14262/2016	14262/16/R	2016	H1N1	1A.3.3.2	EPI2721717
A/swine/Colombia/14266/2016	14266/16/R	2016	H1N1	1A.3.3.2	EPI2721718
A/swine/Colombia/14263/2016	14263/16/R	2016	H1N1	1A.3.3.2	EPI2721719
A/swine/Colombia/14249/2016	14249/16/S	2016	H1N1	1A.3.3.2	EPI2721720

Virus ID corresponds to an internal number assigned by each laboratory plus the year and an acronym of the geographic region where the virus was detected. A: Antioquia, CA: Cauca, CU: Cundinamarca, M: Meta, Q: Quindío, R: Risaralda, and S: Santander. The lineage is presented in the global nomenclature.

**Table 2:** Mean antigenic distance between predicted clusters of swine H1 1A lineage.

	G1A.1	N1A.1	G1A.1–2	N1A.1–3	M1A.3	PDM-CO-16	PDM09-17	PDM-CO-21	PDM-21
G1A.1	0.3 *								
N1A.1	5.2	4.7 *							
G1A.1–2	2.8	6.8	2.6 *						
N1A.1–3	6.2	8.5	6.1	5.0 *					
M1A.3	4.8	8.7	5.4	6.3	1.3 *				
PDM-CO-16	6.5	8.2	6.0	6.0	5.1	0.3 *			
PDM09-17	5.4	7.7	5.0	4.8	5.8	3.6	2.0 *		
PDM-CO-21	6.9	8.3	7.3	4.9	6.3	5.1	3.9	0.2 *	
PDM-21	7.2	8.1	6.8	5.8	7.3	4.5	3.1	3.5	0.9 *

* Mean AD within each cluster.

**Table 3:** Antigenic distance between influenza A viruses of the American 1B cluster according to their phylogenetic and geographic origins.

	Chilean 1B.2-Other	Mexican 1B.2-Other	Asian 1B.2-Other	North America 1B.2.1	North America 1B.2.2	Human
Chilean 1B.2-other	3.1 *					
Mexican 1B.2-other	6.3	0.0 *				
Asian 1B.2-other	4.0	4.5	0.0 *			
North America 1B.2.1	4.2	5.7	3.8	2.9 *		
North America 1B.2.2	5.0	6.3	4.5	4.9	4.0 *	
Human	4.0	3.5	2.1	4.0	4.5	0.2 *

* Antigenic distance between members of the same geographic and phylogenetic origin.

**Table 4:** Epitope conservation among Colombian H1 1A.3.3.2 swine influenza A virus.

Epitope	Conservation	Conserved Amino Acids	Mutations	Associated Region	Associated Year
Sa	38.5%	P124, K153, K154, S157, and P159	G155E	Cundinamarca	2021
			K160M	Antioquia	2021
			K160N	Antioquia	2016
			S162N	Antioquia	2021
				Cundinamarca	
			S162Y	Antioquia	2021
			K163Q	Antioquia	2021
			K163I	Cundinamarca	2021
Sb	50%	D187, Q188, Q189, L191, Y192, and N194	A186T	Cundinamarca	2021
			S190H	Cundinamarca	2021
Ca1	63.3%	N167, K169, G170, S204, and G237	E235D	Antioquia	2021
Ca2	12.5%	G140	P137S	Cundinamarca	2021
				Antioquia	2016
			A141T	Cundinamarca	2021
Cb	50%	L70, T72, and G75	S71F	Antioquia	2016
			A73T	Antioquia	2016
			S74R	Antioquia	2021
				Cundinamarca	

## Data Availability

Genomic data of Colombian viruses analyzed in this study have been published in the GISAID database (https://gisaid.org) and can be consulted under the accession number EPI2719368, EPI2721678, and EPI2721686–EPI2721720.

## Supplementary Materials

The following supporting information can be downloaded at: https://www.mdpi.com/article/10.3390/v15102030/s1, Table S1: Reference strains used in the phylogenetic and antigenic analyses; Table S2: N-Glycosylation motifs predicted in analyzed sequences; Figure S1: Heatmap of the antigenic distance matrix among H1 swine influenza A Virus; Figure S2: Antigenic three-dimensional map of the clusters in the 1A lineage. Figure S3: Antigenic dendrogram and clusters of the H1 1B and 1C lineages of the swine influenza A virus; Figure S4: Antigenic three-dimensional map of the clusters in the 1B and 1C lineage.
