# Supplementary material for: Sequence-Based Antigenic Analyses of H1 Swine Influenza A Viruses from Colombia (2008–2021) Reveals Temporal and Geographical Antigenic Variations
Source: Viruses. 2023 Sep 30;15(10):2030. doi: 10.3390/v15102030 (PMC10612065; doi:10.3390/v15102030)
Supplement: Supplementary file 1 [file viruses-15-02030-s001.zip › Supplementary Material.pdf]

Table S1. Reference strains used in the phylogenetic and antigenic analyses.

Strain	Swine Lineage/Clade	Subtype	Country	Accession number
A/swine/Iowa/15/1930	1A.1	H1N1	USA	ABV25634.1
A/swine/1/Wisconsin/1968	1A.1	H1N1	USA	ABV25636.1
A/swine/Guangxi/12/2005	1A.1	H1N1	China	ADV69039.1
A/swine/Hubei/HG394/2018	1A.1	H1N1	China	QOL21070.1
A/swine/Colombia/0401/2008	1A.1	H1N1	Colombia	AFU83122.1
A/swine/Alberta/SD0154/2016	1A.1.1	H1N1	Canada	ATB53861.1
A/swine/Minnesota/A01781045/2016	1A.1.1	H1N2	USA	AOX49803.1
A/swine/Pennsylvania/A02478616/2019	1A.1.1	H1N2	USA	QEO64518.1
A/swine/Alberta/SD0272/2018	1A.1.1	H1N2	Canada	QAX25196.1
A/swine/Kyoto/3/1979	1A.1-like	H1N1	Japan	BAG49742.1
A/swine/Tochigi/2/2011	1A.1-like	H1N2	Japan	BAM78376.1
A/swine/Korea/CAN01/2004	1A.2	H1N1	Korea	ACE77927.1
A/swine/Kansas/A02245337/2019	1A.2	H1N1	USA	QHF16399.1
A/swine/Texas/A01785906/2019	1A.2-like	H1N1	USA	QBC17636.1
a/swine/Mexico/AVX23/2012	1A.3.1	H1N1	Mexico	AMY15927.1
A/swine/Mexico/GtoDMZC09/2015	1A.3.1	H1N1	Mexico	AVI59052.1
A/swine/Valparaiso/VN1401-559/2014	1A.3.3.2	H1N1	Chile	AYV62774.1
A/swine/Rancagua/VN1401-1107/2015	1A.3.3.2	H1N1	Chile	ARV89702.1
A/California/07/2009	1A.3.3.2	H1N1	USA	YP_009118626.1
A/swine/Illinois/A01493472/2014	1A.3.3.2	H1N1	USA	AHY84443.1
A/swine/Mexico/GtoDMZC04/2015	1A.3.3.2	H1N2	Mexico	AVJ46821.1
A/Jena/VI5258/2009	1A.3.3.2	H1N1	Germany	AGA19175.1
A/swine/UP-India/IVRI01/2009	1A.3.3.2	H1N1	India	AIT38443.1
A/swine/Zambia/264/2018	1A.3.3.2	H1N1	Zambia	BDA36499.1
A/swine/Iowa/A02432387/2019	1A.3.3.2	H1N1	USA	QCQ05164.1
A/Swine/France/53-180028/2018	1A.3.3.2	H1N1	France	AZN23219.1
A/swine/Schallern/IDT19989/2014	1A.3.3.2	H1N1	Germany	ANA11539.1
A/swine/Nuble/VN1401-3960/2018	1A.3.3.2	H1N1	Chile	QDA17083.1
A/swine/South Korea/BRI5_HA/2020	1A.3.3.2	H1N1	Korea	QHW05456.1
A/swine/Zhejiang/SW64/2014	1A.3.3.2	H1N2	China	ALT19800.1
A/swine/Shandong/731/2009	1A.3.3.2	H1N1	China	AEF28990.1
A/pig/Aichi/101/2018	1A.3.3.2	H1N1	Japan	QPF21214.1
A/swine/Japan/KU-YG5/2013	1A.3.3.2	H1N1	Japan	AIT92741.1
A/swine/Colombia/1-01/2009	1A.3.3.2	H1N1	Colombia	AFU83120.1
A/Bogota/WR0090N/2009	1A.3.3.2	H1N1	Colombia	ACY77554.1
A/swine/Antioquia/3-020/2015	1A.3.3.2	H1N1	Colombia	AYD91054.1
A/swine/Ohio/511445/2007	1A.3.3.3	H1N1	USA	ACH69547.1
A/swine/North_Carolina/A01730369/2016	1A.3.3.3	H1N1	USA	AMM43240.1
A/swine/Iowa/19TOSU2739/2019	1A.3.3.3	H1N1	USA	QNH89409.1
A/swine/Scotland/410440/1994	1B.1	H1N2	Scotland	AAD05215.1
A/swine/Bakum/1832/2000	1B.1.2.1	H1N2	Germany	ACR39182.1
A/swine/Italy/60591/2018	1B.1.2.2	H1N2	Italia	AYM94710.1
A/swine/Illinois/A02139356/2018	1B.2.1	H1N2	USA	AVA07303.1
A/swine/Mexico/GtoDMZC01/2014	1B.2.1	H1N2	Mexico	AKQ43522.1
A/swine/Indiana/A02524527/2020	1B.2.1	H1N2	USA	QNL13973.1
A/swine/Alabama/A01104091/2016	1B.2.2.1	H1N2	USA	ANJ61799.1
A/swine/Illinois/A01644323/2018	1B.2.2.1	H1N2	USA	AUS83524.1
A/swine/Iowa/A02524587/2020	1B.2.2.1	H1N2	USA	QOH31678.1

A/swine/South Dakota/A02479076/2020	1B.2.2.2	H1N2	USA	QIP75475.1
A/swine/Chile/VN1401-274/2014	1B.2-other	H1N2	Chile	ARV89358.1
A/swine/Chile/VN1401-4/2014	1B.2-other	H1N1	Chile	ARV89416.1
A/swine/Chile/VN1401-339/2014	1B.2-other	H1N2	Chile	ARV89385.1
A/swine/Mexico/AVX61/2013	1B.2-other	H1N2	Mexico	AMY16231.1
A/swine/Binh Duong/02-16/2010	1B.2-other	H1N2	Vietnam	BAN14742.1
A/swine/O'Higgins/VN1401-4005/2018	1B.2-other	H1N2	Chile	QDA17400.1
A/swine/Finistere/2899/1982	1C.1	H1N1	France	AFR76505.1
A/swine/Belgium/1/1998	1C.2	H1N1	France	ACN67524.1
A/Swine/France/65-150242/2015	1C.2	H1N2	France	QJD66075.1
A/swine/France/22-200113/2020	1C.2	H1N2	France	QUQ73516.1
A/Swine/France/Cotes_d'Armor-0388/2009	1C.2.1	H1N1	France	AGK62667.1
A/swine/Italy/91162/2018	1C.2.1	H1N1	Italy	AYM94721.1
A/swine/Haseluenne/IDT2617/2003	1C.2.2	H1N1	Germany	ACR39185.1
A/swine/China/01/2019	1C.2.3	H1N1	China	QMS51333.1
A/Medellin/WRAIR1297P/2008	Human	H1N1	Colombia	AET84306.2
A/Michigan/45/2015	1A.3.3.2	H1N1	USA	QBL89789.1
A/Brisbane/59/2007	Human	H1N1	Australia	AJK02677.1

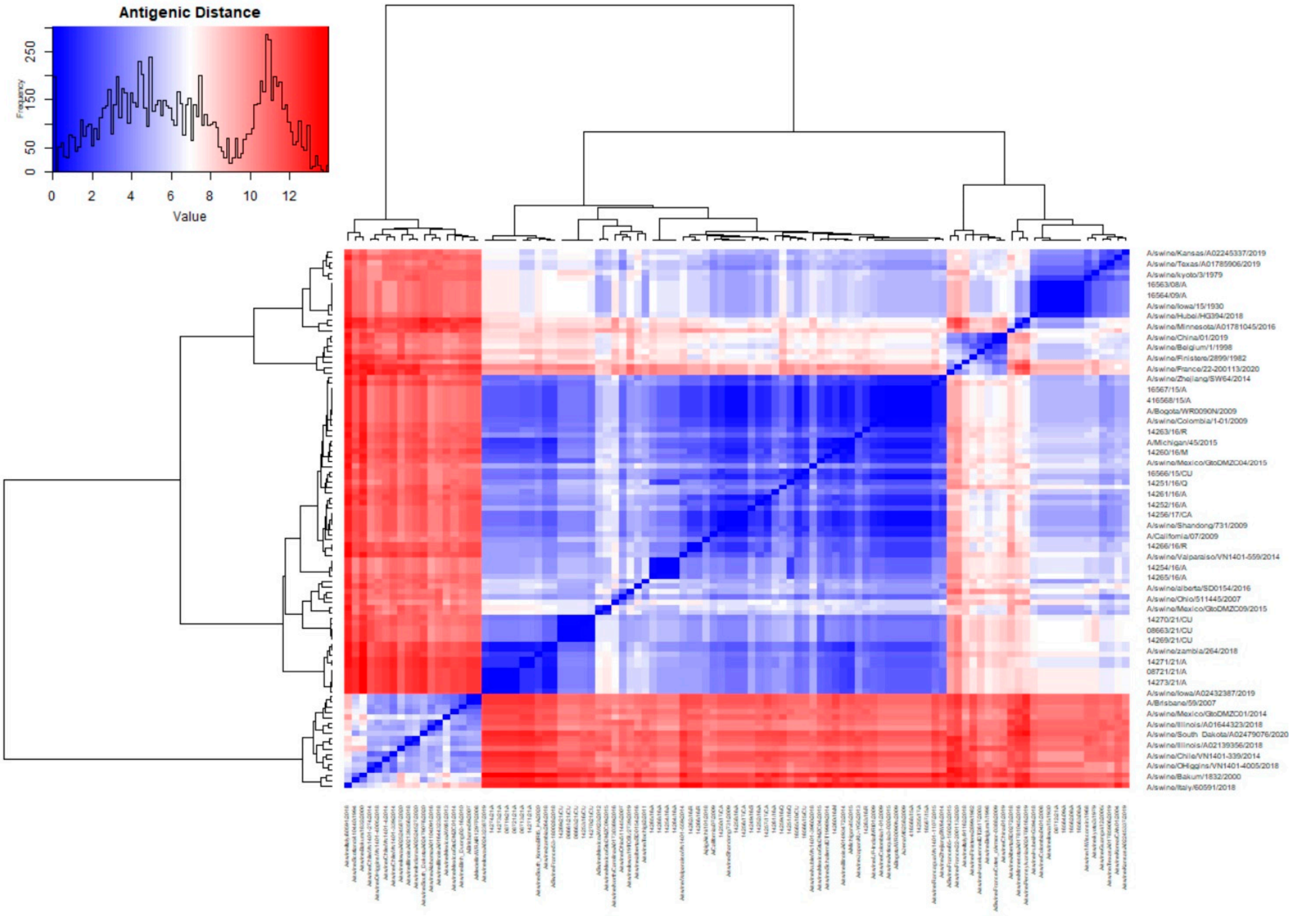

Figure S1. Heatmap of the antigenic distance matrix among H1 Swine Influenza A Virus. The figure shows a symmetric color pattern distributed according to the hierarchical clustering.

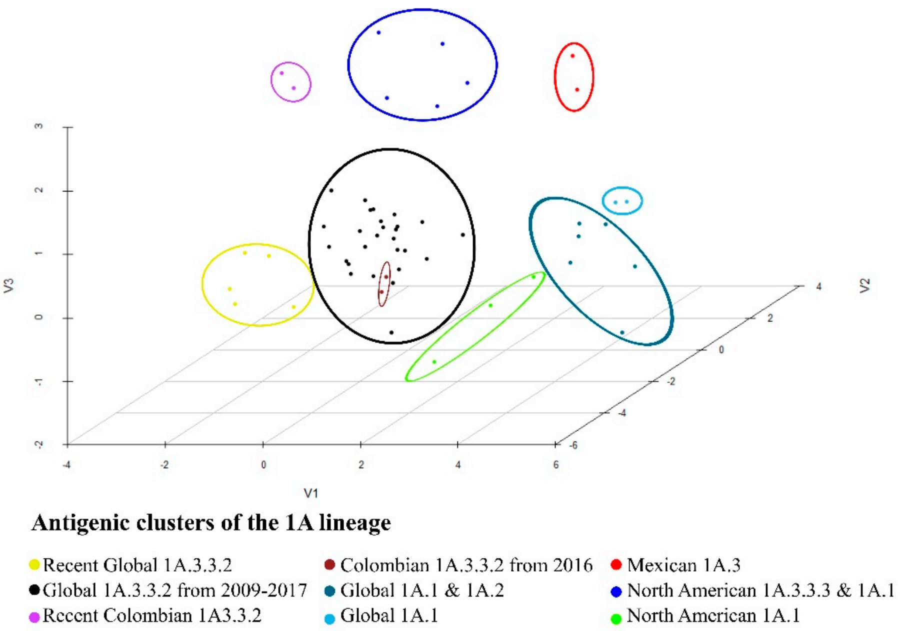

Figure S2. Antigenic three-dimensional map of the clusters in the 1A lineage. The figure shows the antigenic distance existing between the nine calculated antigenic clusters. Each virus was represented as points and colored according to the assigned cluster.

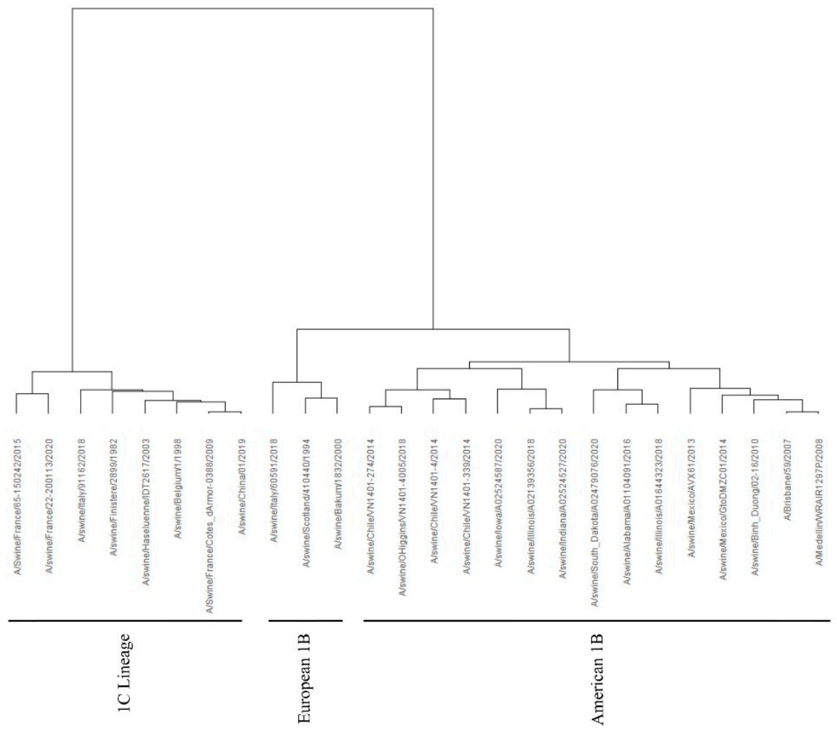

Figure S3. Antigenic dendrogram and clusters of the H1 1B and 1C lineages of the Swine Influenza A virus.

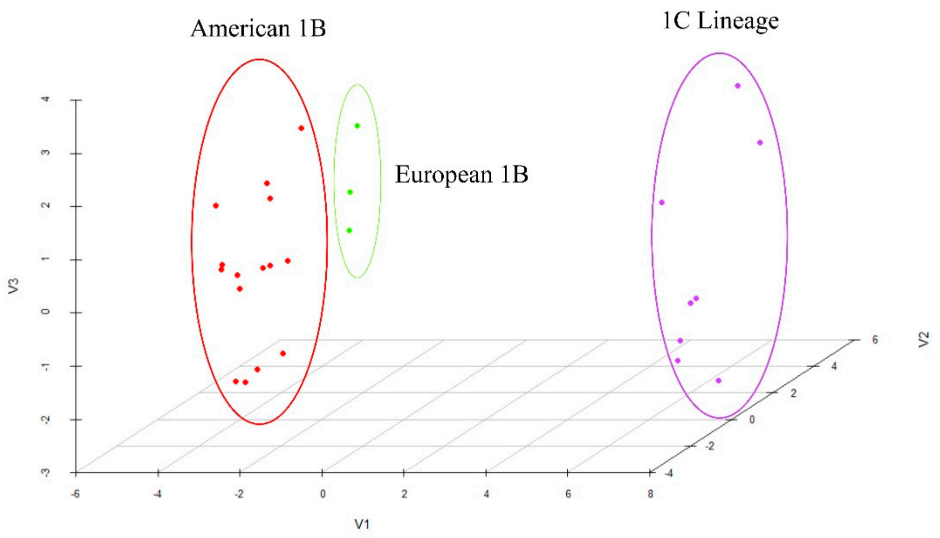

Figure S4. Antigenic three-dimensional map of the clusters in the 1B and 1C lineage. The figure represents the high antigenic distance existence between the 1C and the two 1B clusters. Viruses are presents as points colored according to clustering analysis.

Table S2. N-Glycosylation motifs predicted in analyzed sequences.

Strain	Motifs	Residues
A/swine/Colombia/16562/2008	4	11, 23, 287, 540
A/swine/Colombia/16563/2008	4	11, 23, 287, 540
A/swine/Colombia/16564/2009	4	11, 23, 287, 540
A/swine/Colombia/16567/2015	4	11, 23, 287, 540
A/swine/Colombia/16568/2015	4	11, 23, 287, 540
A/swine/Colombia/14254/2016	5	11, 23, 160, 287, 540
A/swine/Colombia/14258/2016	4	11, 23, 287, 540
A/swine/Colombia/14264/2016	5	11, 23, 160, 287, 540
A/swine/Colombia/14268/2016	5	11, 23, 160, 287, 540
A/swine/Colombia/14252/2016	4	11, 23, 287, 540
A/swine/Colombia/14265/2016	5	11, 23, 160, 287, 540
A/swine/Colombia/14261/2016	4	11, 23, 287, 540
A/swine/Colombia/14255/2017	4	11, 23, 287, 540
A/swine/Colombia/08712/2021	4	11, 23, 287, 540
A/swine/Colombia/08713/2021	4	11, 23, 287, 540
A/swine/Colombia/14271/2021	4	11, 23, 287, 540
A/swine/Colombia/08719/2021	5	11, 23, 162, 287, 540
A/swine/Colombia/08721/2021	5	11, 23, 162, 287, 540
A/swine/Colombia/14273/2021	5	11, 23, 162, 287, 540
A/swine/Colombia/14274/2021	5	11, 23, 162, 287, 540
A/swine/Colombia/14250/2017	4	11, 23, 287, 540
A/swine/Colombia/14256/2017	4	11, 23, 287, 540
A/swine/Colombia/14257/2017	4	11, 23, 287, 540
A/swine/Colombia/16565/2010	4	11, 23, 287, 540
A/swine/Colombia/16566/2015	4	11, 23, 287, 540
A/swine/Colombia/14253/2016	4	11, 23, 287, 540

A/swine/Colombia/08661/2021	3	11, 23, 240
A/swine/Colombia/08663/2021	3	11, 23, 240
A/swine/Colombia/14269/2021	3	11, 23, 240
A/swine/Colombia/14270/2021	3	11, 23, 240
A/swine/Colombia/14260/2016	4	11, 23, 287, 540
A/swine/Colombia/14259/2016	4	11, 23, 287, 540
A/swine/Colombia/14251/2016	4	11, 23, 287, 540
A/swine/Colombia/14262/2016	4	11, 23, 287, 540
A/swine/Colombia/14266/2016	4	11, 23, 287, 540
A/swine/Colombia/14263/2016	4	11, 23, 287, 540
A/swine/Colombia/14249/2016	4	11, 23, 287, 540
